# Disseminated mucocutaneous trichosporonosis in a patient with histiocytic sarcoma^☆^^☆☆^

**DOI:** 10.1016/j.abd.2021.01.003

**Published:** 2021-07-18

**Authors:** Arturo Robles-Tenorio, Rossy Anahí Rivas-López, Alexandro Bonifaz, Víctor Manuel Tarango-Martínez

**Affiliations:** a Instituto Dermatológico de Jalisco “Dr. José Barba Rubio”, Zapopan, Jalisco, Mexico; b Centro Dermatológico de Sinaloa "Dr. Jesús Rodolfo Acedo Cárdenas", Culiacán, Sinaloa, México; c Servicio de dermatología y departamento de Micología, Hospital General de México “Dr. Eduardo Liceaga”, Ciudad de México, México

**Keywords:** Hematologic neoplasms, Histiocytic sarcoma, Trichosporon, Trichosporonosis, Ulcer

## Abstract

*Trichosporon asahii* is the causal agent of trichosporonosis. Patients with immunosuppression or hematological malignancies are at higher risk of infection. Skin and mucosal involvement appear as fast-growing papulonodular lesions and necrotic ulcers. Internal organ dissemination is lethal. Therapeutic success depends on the underlying disease. Here, the authors present the first case of disseminated mucocutaneous trichosporonosis in a patient with a post-mortem diagnosis of histiocytic sarcoma, a rare and aggressive haematolymphoid neoplasm. Regretfully, death occurred despite treatment with liposomal amphotericin B and supportive measures, showcasing the fatality of both diseases.

## Case report

**Figure 1:**
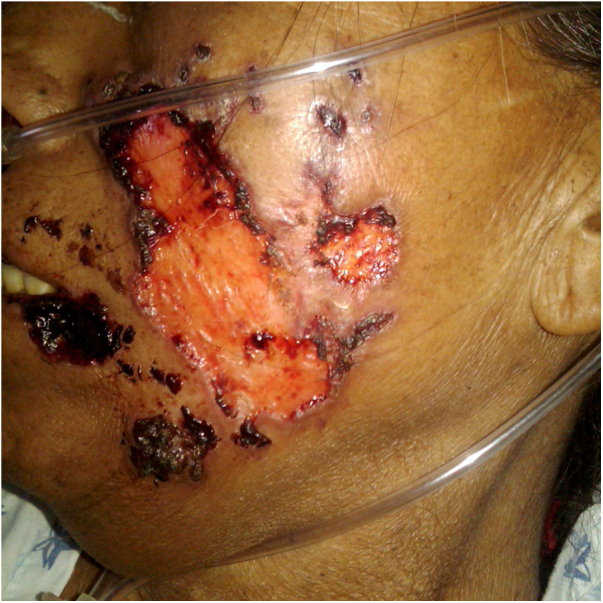
Large, necrotic face ulcers affecting the cheek and the oral mucosa.

**Figure 2:**
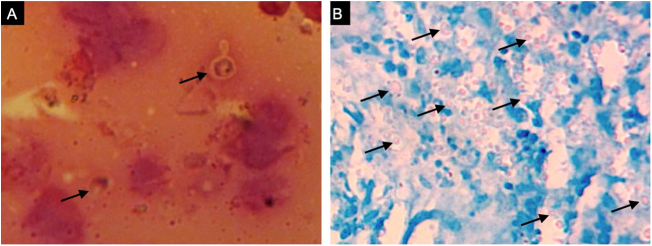
Face ulcer examinations. (A), Budding yeast cells and (B), PAS stained blastoconidia are observed on a Gram smear and on histopathology, respectively.

**Figure 3:**
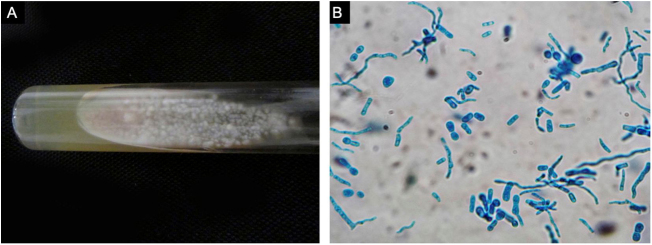
Face ulcer culture. (A), *Trichosporon spp.* colonies showed a raised, waxy appearance with radial furrows on Sabouraud culture. (B), Culture smear revealed hyphae, blastoconidia and arthroconidia with cotton blue stain.

A previously healthy 57-year-old indigenous woman presented to the clinic with an 18-month history of facial wounds, progressive pleuritic pain, dyspnea, productive cough, weight loss, fever, and fatigue. On examination, there were multiple necrotic ulcers on the left cheek, nose, and lips (Fig. 1). Submaxillary, cervical, and axillary lymph nodes appeared swollen. Laboratory results showed anemia (4.6 g/dL) and LDH elevation (1477 UI/L). Serologies for HIV 1/2, Hepatitis B and C were negative. Computed tomography exhibited basal, bilateral consolidations and atelectasis, hepatomegaly, and free intraperitoneal fluid. Face ulcer smear and histopathology reported budding yeast cells and blastoconidia (Fig. 2). Despite receiving liposomal amphotericin B, the patient presented lesions dissemination to the oral cavity, lower gastrointestinal bleeding, mixed shock, and died fifteen days after admission. Face ulcer culture (Fig. 3) and axillary lymph node histopathology were collected post-mortem. *Trichosporon asahii* was identified by matrix-assisted laser desorption ionization-time of flight mass spectrometry (MALDI-TOF MS, Vitek-MS®). Axillary node histopathology showed Histiocytic Sarcoma (HS), with positive CD68 and CD163 on immunohistochemistry.

## Discussion

*Trichosporon* spp. are found as commensal and pathogenic fungi, capable of evading the immune response and generating antimicrobial resistance through biofilm formation, metabolic, and phenotypic features.^1^ *Trichosporon asahii* affects the skin, mucous membranes, and internal viscera of patients with immunosuppression, hematolymphoid malignancies, or chemotherapy-induced neutropenia.^2^ Disseminated mucocutaneous trichosporonosis presents as rapidly progressive papulonodular lesions and necrotic ulcers, similar to cryptococcosis.^3^

Trichosporonosis is suspected by observing hyphae, arthroconidia, and blastoconidia on a smear and histopathology.^3^ The definitive diagnosis relies on species identification from culture colonies by chromogenic or biochemical methods.^2^, ^4^ MALDI-TOF MS might soon replace these processes, providing higher accuracy yields.^5^ Other techniques such as flow cytometry, polymerase chain reaction, and gene sequencing are still under investigation.^4^

Comparative evidence of antifungal therapy is limited. In a systematic review of 203 cases, voriconazole had the highest favorable outcome rate (73.6%) in patients with hematological neoplasms, and also the best *in vitro* activity against *Trichosporon spp.* Similarly, in Mexico, minimum inhibitory concentrations were the lowest for triazoles, higher for amphotericin B, and the highest for echinocandins.^6^ Together, these results are in line with treatment guidelines, where voriconazole is the preferred antifungal. In the present case study, the authors used liposomal amphotericin B due to availability at the authors’ institution.^2^, ^4^

Therapeutic success depends on the underlying disease status. Here, the patient had an advanced case of HS. This rare and aggressive cancer represents 1% of all hematolymphoid neoplasms.^7^ HS usually affects lymph nodes, but it can speedily disseminate to several organs. On histopathology, the tumor shows diffuse proliferation of neoplastic cells with eosinophilic cytoplasm and eccentric nuclei. Immunohistochemistry is positive for CD163, CD68, and lysozyme. Epithelial, melanocytic, myeloid, Langerhans, B, and T cell markers are negative. As in this patient, HS is often a late diagnosis and has an estimated survival of fewer than two years.^7^, ^8^

In conclusion, trichosporonosis and HS are infrequent, clinically challenging diseases. A swift, interdisciplinary action between dermatology, oncology, and infectious diseases specialists is of utmost importance.

## Financial support

None declared.

## Authors’ contributions

Arturo Robles-Tenorio: Critically reviewed the literature, analyzed the data, and wrote the final version of the manuscript.

Rossy Anahí Rivas-López: Prepared the original draft, was responsible of study conception and data collection.

Alexandro Bonifaz: Critically reviewed the manuscript, provided supporting references, and approved the final version of the manuscript.

Víctor Manuel Tarango-Martínez: Prepared the original draft, collected the data, and approved the final version of the manuscript.

## Conflicts of interest

None declared.
